# Optimal strength and timing of steroids in the management of erlotinib-related skin toxicities in a post-marketing surveillance study (POLARSTAR) of 9909 non-small-cell lung cancer patients

**DOI:** 10.1007/s10147-015-0893-5

**Published:** 2015-10-26

**Authors:** Naoya Yamazaki, Yoshio Kiyohara, Shoji Kudoh, Akihiro Seki, Masahiro Fukuoka

**Affiliations:** Department of Dermatologic Oncology, National Cancer Center Hospital, 5-1-1 Tsukiji, Chuo-Ku, Tokyo, 104-0045 Japan; Dermatology Division, Shizuoka Cancer Center, Shimonagakubo, Nagaizumi-cho, Sunto-Gun, Shizuoka, 411-8777 Japan; Double-Barred Cross Hospital Japan Anti-Tuberculosis Association, Matsuyama, Kiyose-Shi, Tokyo, 204-8522 Japan; Chugai Pharmaceutical Co. Ltd, Nihonbashi-Muromachi, Chuo-Ku, Tokyo, 103-8324 Japan; Izumi Municipal Hospital, Fuchu-cho, Izumi-Shi, Osaka, 594-0071 Japan

**Keywords:** Non-small-cell lung cancer, Erlotinib, Japanese, Rash management, Steroids

## Abstract

**Background:**

Skin toxicities, such as rash, are the most common adverse reactions associated with erlotinib. Steroids are a key treatment option for rash management; however, optimal timing of administration and selection of steroid strength have not been fully established. In this surveillance study of Japanese non-small-cell lung cancer (NSCLC) patients treated with erlotinib, rash management using topical steroids was analyzed in routine clinical practice.

**Methods:**

From December 2007 to October 2009, all recurrent/advanced NSCLC patients in Japan treated with erlotinib were enrolled into this study (POst-Launch All-patient Registration Surveillance in TARceva). The observation period was 12 months, and data for all adverse events were collected. Erlotinib-related rash, interventions for the symptoms, and outcomes of the interventions were analyzed.

**Results:**

A total of 9909 patients were evaluated. Rash occurred in 67.4 % of patients; grade 1, 2, and 3 rash were observed in 26.8 %, 32.4 %, and 7.2 % of patients, respectively. The most common management strategy was topical steroids in 75.0 % of patients with rash. Regardless of rash grade, earlier initiation of steroids resulted in quicker recovery. In those for whom topical steroids were initiated more than 21 days after rash onset, median recovery time was more than 100 days regardless of rash grade, compared with those treated before rash onset, whose median time to recovery was 35–51 days, depending on rash grade. Median time to recovery of rash in the group initiated on medium-rank steroids then changed to strong-rank steroids was 47, 98, and 103 days for those with grade 1, 2, and 3 rash, respectively, compared with 39, 53, and 73 days median recovery for grade 1, 2, and 3 rash, respectively, in patients initiated on strong-rank steroids.

**Conclusion:**

Earlier initiation of topical steroids for the management of rash with strong or higher-rank steroids could achieve faster improvement.

**Electronic supplementary material:**

The online version of this article (doi:10.1007/s10147-015-0893-5) contains supplementary material, which is available to authorized users.

## Introduction

Erlotinib is an orally administered epidermal growth factor receptor (EGFR) tyrosine-kinase inhibitor (TKI). The phase III BR.21 study showed that erlotinib treatment of non-small-cell lung cancer (NSCLC) in the second- or third-line setting achieved significant overall survival (OS), progression-free survival (PFS), and response rate benefit compared with best supportive care [1]. In addition, the OPTIMAL and EURTAC studies have reported significant PFS benefits with erlotinib as first-line treatment for patients with *EGFR* mutation-positive NSCLC compared with chemotherapy in Asian and European populations, respectively [2, 3].

Skin toxicities (especially acneiform rash) are the most common adverse reactions associated with erlotinib treatment [4, 5]. Across NSCLC phase III studies, the incidence of rash is 62–76 % [6]. This finding is not unexpected as EGFR is expressed in undifferentiated and proliferating keratinocytes of the skin, meaning that EGFR TKIs often result in skin toxicity [7]. The most common erlotinib-related skin toxicities are acneiform rash, xeroderma, paronychia, and pruritus [8–10].

The incidence of skin rash in Japanese patients treated with erlotinib has been high, with up to 98.1 % of patients experiencing rash [8]. In phase II studies, 72.2 % of Japanese patients experienced xeroderma, which is characterized by dry, rough skin causing fissures and a scaling effect [9]. Pruritus, or skin itching, is common with the development of rash or xeroderma. Paronychia is a painful erythema around several fingernails or toenails, which can result in swelling, granulation, and bleeding.

Although most cases of skin toxicities are mild and transient, they can have a considerable impact on patients’ quality of life and can therefore reduce compliance with erlotinib therapy. A number of studies have reported evidence suggesting a correlation between the incidence and severity of rash with improved clinical outcomes, such as longer OS among erlotinib-treated patients [11–13]. In the BR.21 study, all grades of rash were associated with longer OS compared with patients who did not develop rash [grade 1 rash vs. no rash: hazard ratio (HR) 0.41; *P* < 0.001; grade ≥2 rash vs. no rash: HR 0.29; *P* < 0.001] [13]. An association between rash and prolonged OS has also been reported in Japanese patients (OS 8.8 months for patients with no rash compared with 16.6 months for patients with grade 2/3 rash) [14]. Considering the correlation between rash and survival outcomes, adequate rash management (prophylactic cleansing regimens, reducing the dose or interruption of erlotinib treatment, or use of concomitant treatment for rash) is of the utmost importance to ensure the continuation of erlotinib treatment and therefore the maximum benefit for patients.

Kiyohara et al. developed an algorithm for rash management (i.e., treatment course for rash symptoms) consisting of the use of strong or higher-class steroids to ‘manage’ rash, allowing patients to continue erlotinib use [15]. The use of steroids is an option for rash management depending on the grade of rash. In Japan, a five-class ranking system for steroids is used ranging from the strongest to very strong, strong, medium, and weak. It is generally advised that only strong or higher-potency steroids are used to treat grade ≥2 erlotinib-related rash [15]. Topical steroids (strong or higher rank) for grade ≥2 toxicities are recommended to treat both xeroderma and pruritus. Strong or higher-rank steroids are recommended for the treatment of paronychia.

This analysis was part of the POst-Launch All-patient Registration Surveillance in TARceva (POLARSTAR) study [10]. POLARSTAR is a large-scale surveillance study undertaken as a post-approval commitment to monitor the efficacy and safety of erlotinib in Japan. This current analysis evaluates the use of topical steroids as a treatment for rash. The frequency of skin toxicity-related adverse events (AEs), the interventions used, and their outcomes were analyzed.

## Methods

### Study design

In this phase IV observational study, all patients with unresectable, recurrent, or advanced NSCLC who were treated with erlotinib were enrolled. The study was approved by the relevant ethics committees.

### Treatment schedule

Patients receiving erlotinib daily were monitored until termination of erlotinib therapy or completion of 12 months of treatment. Erlotinib treatment delay, dose reduction, and discontinuation were permitted in actual clinical practice to manage rash.

### Assessments

Demographic and baseline data were collected for each patient, including age, gender, body mass index, tumor histology, Eastern Cooperative Oncology Group (ECOG) performance status (PS), smoking history, and medical history (including hepatic dysfunction, renal dysfunction, cardiovascular disease, and lung disorders). Safety data were collected at 1, 6, and 12 months after the start of erlotinib therapy. All AE reports were collected, and AEs were graded using the National Cancer Institute Common Terminology Criteria for AEs version 3.0 and coded using the Medical Dictionary for Regulatory Activities version 14.1 thesaurus terms.

### Outcome measures

Frequency of erlotinib-related skin toxicities (acneiform rash, xeroderma, pruritus, and paronychia), interventions for the symptoms, and the outcomes of these interventions were assessed by time to treatment initiation, recovery rate, and time to recovery. To avoid confounding factors, only the first event of skin toxicity was analyzed. For rash management interventions, standard Japanese ranking of steroids was used, defining steroids as strongest (e.g., clobetasol propionate), very strong (e.g., dexamethasone propionate), strong (e.g., betamethasone valerate), medium (e.g., hydrocortisone butyrate), and weak (e.g., hydrocortisone acetate) [16]. Patients were categorized into subgroups according to the topical steroid treatment they received (weak- or medium-rank steroids categorized as ‘medium,’ strong, or higher-rank steroids categorized as ‘strong,’ and those initiated on medium-rank or lower steroids then changed to strong-rank or higher-rank steroids were categorized as ‘medium to strong’). Time to recovery was estimated from Kaplan–Meier curves.

## Results

### Patients

A total of 10,708 patients were enrolled between December 2007 and October 2009 from 1027 institutions; of these, 9,909 patients were evaluated for this analysis. Baseline characteristics are shown in Table 1. Briefly, the median patient age was 66 years; the majority of patients (80.2 %) had adenocarcinoma histology; 44 % of patients had an ECOG PS of 1; and 29.7 % of patients had ECOG PS of 0.

**Table 1:** Baseline characteristics of the analysis population (*N* = 9909)

	*N*	%
Gender		
Male	5300	53.5
Female	4609	46.5
Age (years)		
Median (range)	66 (14–95)	–
Histology		
Adenocarcinoma	7950	80.2
Other	1935	19.5
Unknown	24	0.24
Stage		
Relapsed	3316	33.5
IIIB	1387	14.0
IV	4917	49.6
Other	202	2.0
Unknown	87	0.9
ECOG PS		
0	2935	29.6
1	4380	44.2
2	1786	18.0
3	604	6.1
4	186	1.9
Unknown	18	0.2

*ECOG PS* Eastern Cooperative Oncology Group performance status

### Incidence of skin toxicity

The most common skin toxicities were acneiform rash, xeroderma, and paronychia, observed in 60.9 %, 7.5 %, and 6.6 % of the study population, respectively. The majority of these skin toxicities were mild in severity, as grade 3/4 acneiform rash, xeroderma, and paronychia were reported in only 6.3 %, 0.3 %, and 0.7 % of patients, respectively (Table 2). Three grade 5 skin toxicities reported: one case of toxic skin eruption and two cases of Stevens–Johnson syndrome.

**Table 2:** Erlotinib-related skin toxicities by grade (*N* = 9909)

*N* (%)	Grade 1	Grade 2	Grade 3	Grade 4	Grade 5	Total
Acneiform rash	2415 (24.4)	2944 (29.7)	598 (6.0)	23 (0.2)	1 (0.0)	6032 (60.9)
Xeroderma	422 (4.3)	286 (2.9)	24 (0.2)	1 (0.0)	0	738 (7.5)
Paronychia	274 (2.8)	303 (3.1)	70 (0.7)	0	0	654 (6.6)

The median time from erlotinib administration to onset of acneiform rash was within 2 weeks (9 days), xeroderma was within 3 weeks (16 days), and paronychia was approximately 5 weeks (34 days) from initial erlotinib administration.

### Interventions for skin toxicity

The most common intervention for the treatment of skin toxicities was topical steroids, with more than 75 % of patients who suffered from acneiform rash (the most common skin toxicity) receiving steroids within 4 days of diagnosis. Of the patients experiencing xeroderma, more than 75 % received steroids within 5 days of onset or diagnosis, and many patients with paronychia were already on steroids, for an average of 10 days, before diagnosis or onset of paronychia.

**Fig. 1:**
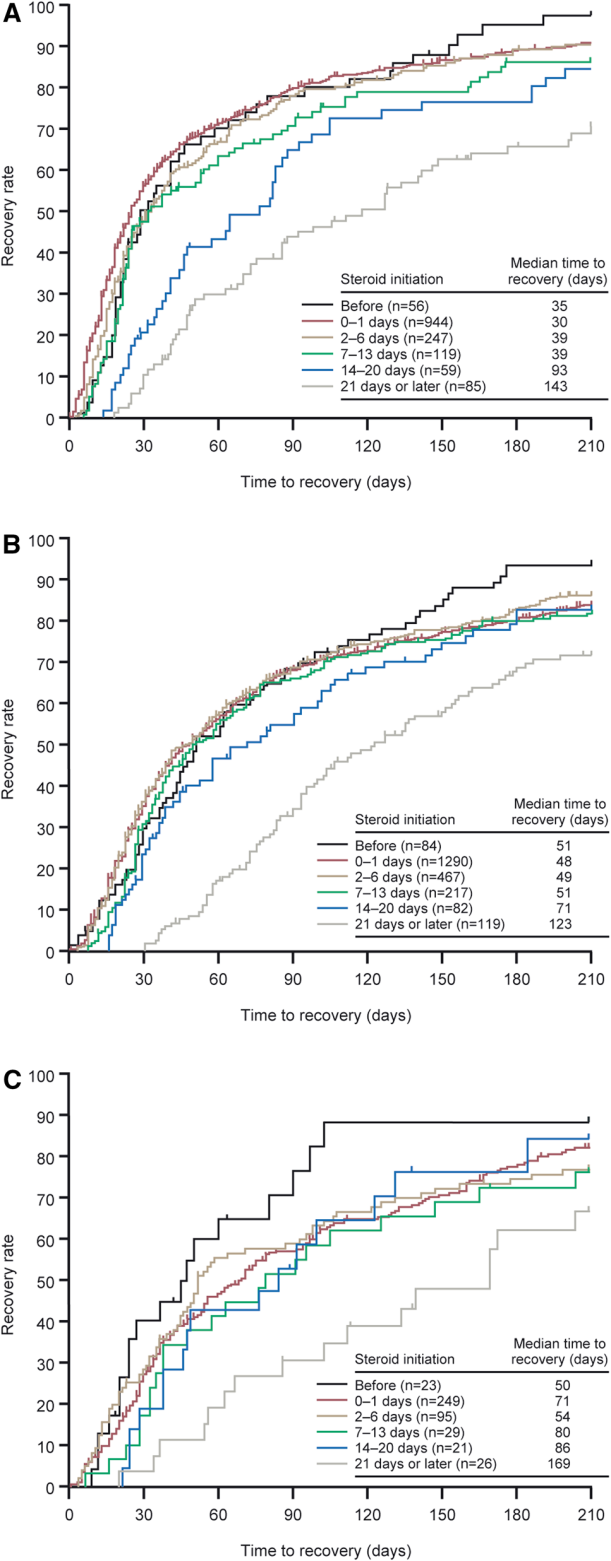
Time to recovery according to time to treatment initiation: grade 1 rash (**a**), grade 2 rash (**b**), grade ≥3 rash (**c**)

Regardless of rash grade, earlier initiation of topical steroids resulted in quicker recovery (Fig. 1). Although the early initiation groups (before onset, 0–1, 2–6, and 7–13 days) had similar median recovery times (30–39 days depending on initiation group for grade 1 rash, 48–51 days depending on initiation group for grade 2 rash), there was a noticeable increase in recovery time from the 14–20 days group (93 days for grade 1 rash, 71 days for grade 2 rash). In the group initiated more than 21 days after onset, recovery appeared to be considerably longer than the other groups, resulting in a recovery time of more than 100 days regardless of rash grade, compared with the before onset groups who had median time to recovery of 35–51 days depending on rash grade.

**Fig. 2:**
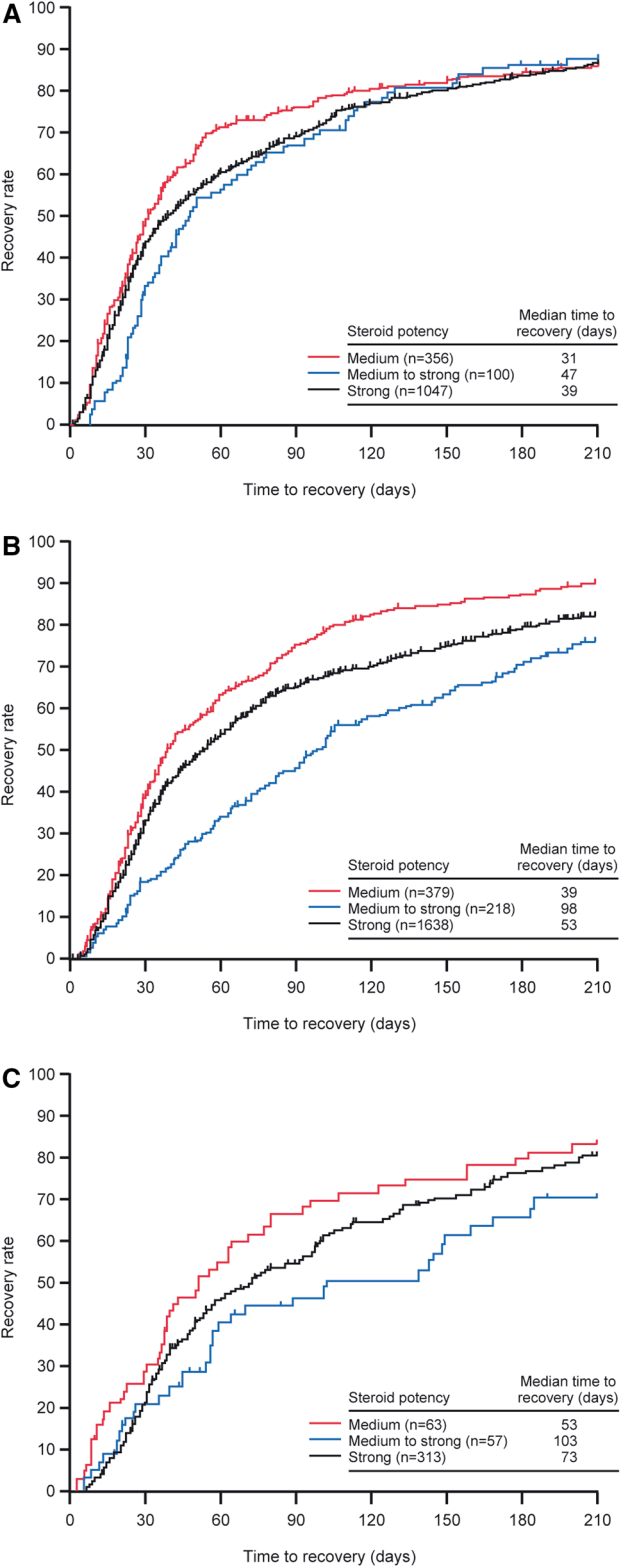
Class effects of steroids for rash management: grade 1 rash (**a**), grade 2 rash (**b**), and grade ≥3 rash (**c**). Medium: patients treated with medium- or weak-rank steroids; medium to strong: patients initially treated with medium- or weak-rank steroids then changed to strong- or higher-rank steroids; strong: patients treated with strong- or higher-rank steroids

Median time to recovery stratified by steroid strength and by grade of rash is shown in Fig. 2. Time to recovery in patients who were changed to strong- or higher-rank steroids (medium to strong) was longer than that observed in other groups. Median time to recovery of rash in the medium- to strong-rank steroids subgroup was 47, 98, and 103 days for those with grade 1, 2, and 3 rash, respectively; this is compared with 39, 53, and 73 days median recovery for grade 1, 2, and 3 rash, respectively, in patients in the strong-rank steroid subgroup. In patients who received medium-rank steroids for grade 1, 2, or 3 rash, 21.9 %, 36.5 %, and 47.5 % of patients, respectively, needed to have their treatment changed to strong-rank steroids.

The trend of patients in the medium to strong subgroup having longer recovery time than those responding to medium-rank steroids or those initiated on strong-rank steroids was also seen in patients who did not have erlotinib dose reduction or interruption (Supplementary Fig. 1).

In patients with grade 2 rash (the most common grade of rash), there was again a trend of patients in the medium to strong subgroup having longer recovery times than the other two groups, regardless of time of steroid initiation (Supplementary Fig. 2). In patients with grade 2 rash, there was a trend of earlier initiation of steroid, resulting in shorter recovery time, regardless of steroid rank.

## Discussion

This analysis focused on the incidence and management of rash in POLARSTAR, as skin toxicities are one of the most common AEs associated with erlotinib, with rash being the most common skin toxicity experienced. As rash can have an impact on patients’ quality of life and may lead to discontinuation of treatment, effective rash management is required to ensure patients remain on erlotinib for as much of the treatment course as possible to gain maximal benefit. As erlotinib treatment is often given for an extended period of time, particularly in patients with *EGFR* mutations, the development of effective rash management strategies to ameliorate ongoing rash symptoms is vital, particularly as prophylactic rash treatments have not yet been fully validated. Effective management strategies are especially important when considering that some data indicate a correlation between increased rash grade and erlotinib efficacy, meaning those with the most severe rash, who may wish to discontinue treatment, may actually be gaining the most benefit from erlotinib [11–14].

The majority of skin toxicities reported in the POLARSTAR Japanese surveillance study were grade 1/2. Acneiform rash was the most common skin toxicity observed, seen in 60.9 % of patients. Earlier initiation of topical steroid treatment for erlotinib-related rash resulted in reduced recovery time. Patients who were initiated on strong-rank steroids had a shorter recovery time than patients who failed to respond to medium-rank steroids and then progressed to strong-rank steroids, regardless of whether patients received additional erlotinib dose reduction or interruption for rash. These data suggest that earlier initiation of treatment (within 0–14 days of diagnosis) with strong-ranked or higher-rank steroids could be a suitable administration regimen for rash management in erlotinib-treated NSCLC patients. Patients who were initiated on medium-rank steroids had the shortest recovery time; however, some patients needed to change steroid rank (21.9 % of patients with grade 1 rash, 36.5 % with grade 2 rash, and 47.5 % with grade 3 rash needed to change from medium- or weaker-rank steroids to stronger-rank steroids). Furthermore, patients who needed to change steroid rank had the longest recovery time. This finding suggests there may be some risk of undertreatment with medium-rank steroids because it is difficult to determine precisely whether medium-rank steroids have enough intensity for each case before treatment initiation. Additionally, undertreatment with medium-rank steroids could lead to longer recovery time than initiation with strong-rank steroids. Therefore, if there is no adequate reason for avoiding administration of strong-rank steroids (e.g., concomitant skin infection), it might be more effective to initiate all patients on strong- or higher-rank steroids for maximal benefit and quicker recovery time.

There are various ways to manage erlotinib-related rash. A recent review by Kiyohara et al. highlighted very strong/strong class steroids as a recommended treatment for EGFR-related acneiform rash [15]. In addition to steroids ranging from hydrocortisone to methylprednisolone for varying grades of rash, patient education is also seen as important for prophylactic treatment (teaching patients about moisturization, reducing sun exposure, and avoiding products that dry the skin) [6]. Novel treatments, such as menadione lotion, retinoids, and alpha-hydroxy acids, are also being investigated as possible treatment options for erlotinib-related rash [6]. Earlier steroid treatments for skin toxicities and even pre-emptive regimens have been effective in reducing EGFR TKI-related rash. Lacouture et al. showed that pre-emptive steroid treatment reduced the incidence of grade ≥2 skin toxicities by 50 % compared with reactive treatment in colorectal cancer patients treated with panitumumab [29 % vs. 62 % of patients: odds ratio 0.3; 95 % confidence interval (CI), 0.1–0.6] [17]. However, these novel pre-emptive treatments are neither fully established nor validated; therefore, adequate reactive steroid treatment is still a key management strategy in the current scenario.

To our knowledge, this is the first analysis focusing on the correlation between steroid rank, timing of initiation of steroid treatment, and recovery time of rash induced by erlotinib. However, there are a number of factors to consider when interpreting data from this analysis. As this was a single-arm surveillance study, there was no control group with which to directly compare results. The study design meant that in contrast to a clinical trial, there was no strict observation period, and the study lacked any patient selection criteria, as all patients treated with erlotinib in Japan in the post-approval period were enrolled.

## Conclusion

As most current treatment algorithms are based on anecdotal evidence, and this study provides evidence to support the use of topical steroids for EGFR TKI-associated rash, further studies should be undertaken to corroborate our findings that strong topical steroids initiated early in skin toxicity diagnosis are a suitable regimen to treat these AEs to allow continuation of EGFR TKI therapy.

## Electronic supplementary material

Below is the link to the electronic supplementary material.

Supplementary Fig. S1 Class effects of steroids for rash management in patients who did not have an erlotinib dose reduction or interruption: grade 1 rash (**a**), grade 2 rash (**b**), and grade ≥ 3 rash (**c**). Medium: patients treated with medium- or weak-rank steroids; medium to strong: patients initially treated with medium- or weak-rank steroids then changed to strong- or higher-rank steroids; strong: patients treated with strong- or higher-rank steroids. (DOCX 13 kb)

Supplementary Fig. S2 Time to recovery by rank of steroid and time to treatment initiation in patients with grade 2 rash. (TIFF 1237 kb)

Supplementary material 3 (TIFF 1409 kb)
