# Supplementary material for: Optimal strength and timing of steroids in the management of erlotinib-related skin toxicities in a post-marketing surveillance study (POLARSTAR) of 9909 non-small-cell lung cancer patients
Source: Int J Clin Oncol. 2015 Oct 26;21:248–53. doi: 10.1007/s10147-015-0893-5 (PMC4824834; doi:10.1007/s10147-015-0893-5)
Supplement: Supplementary file 1 — Supplementary Fig. S1 Class effects of steroids for rash management in patients who did not have an erlotinib dose reduction or interruption: grade 1 rash (a), grade 2 rash (b), and grade ≥ 3 rash (c). Medium: patients treated with medium- or weak-rank steroids; medium to strong: patients initially treated with medium- or weak-rank steroids then changed to strong- or higher-rank steroids; strong: patients treated with strong- or higher-rank steroids. (DOCX 13 kb) [file 10147_2015_893_MOESM1_ESM.docx]

**SUPPLEMENTARY DATA**

**Supplementary Fig. S1** Class effects of steroids for rash management in patients who did not have an erlotinib dose reduction or interruption (A) grade 1 rash, (B) grade 2 rash, and (C) grade ≥3 rash. Medium: patients treated with medium- or weak-rank steroids; Medium to strong: patients initially treated with medium- or weak-rank steroids then changed to strong- or higher-rank steroids; Strong: patients treated with strong- or higher-rank steroids

**Supplementary Fig. S2** Time to recovery by rank of steroid and time to treatment initiation in patients with grade 2 rash
