# Supplementary figures and images for: Optimal strength and timing of steroids in the management of erlotinib-related skin toxicities in a post-marketing surveillance study (POLARSTAR) of 9909 non-small-cell lung cancer patients
Source: Int J Clin Oncol. 2015 Oct 26;21:248–53. doi: 10.1007/s10147-015-0893-5 (PMC4824834; doi:10.1007/s10147-015-0893-5)

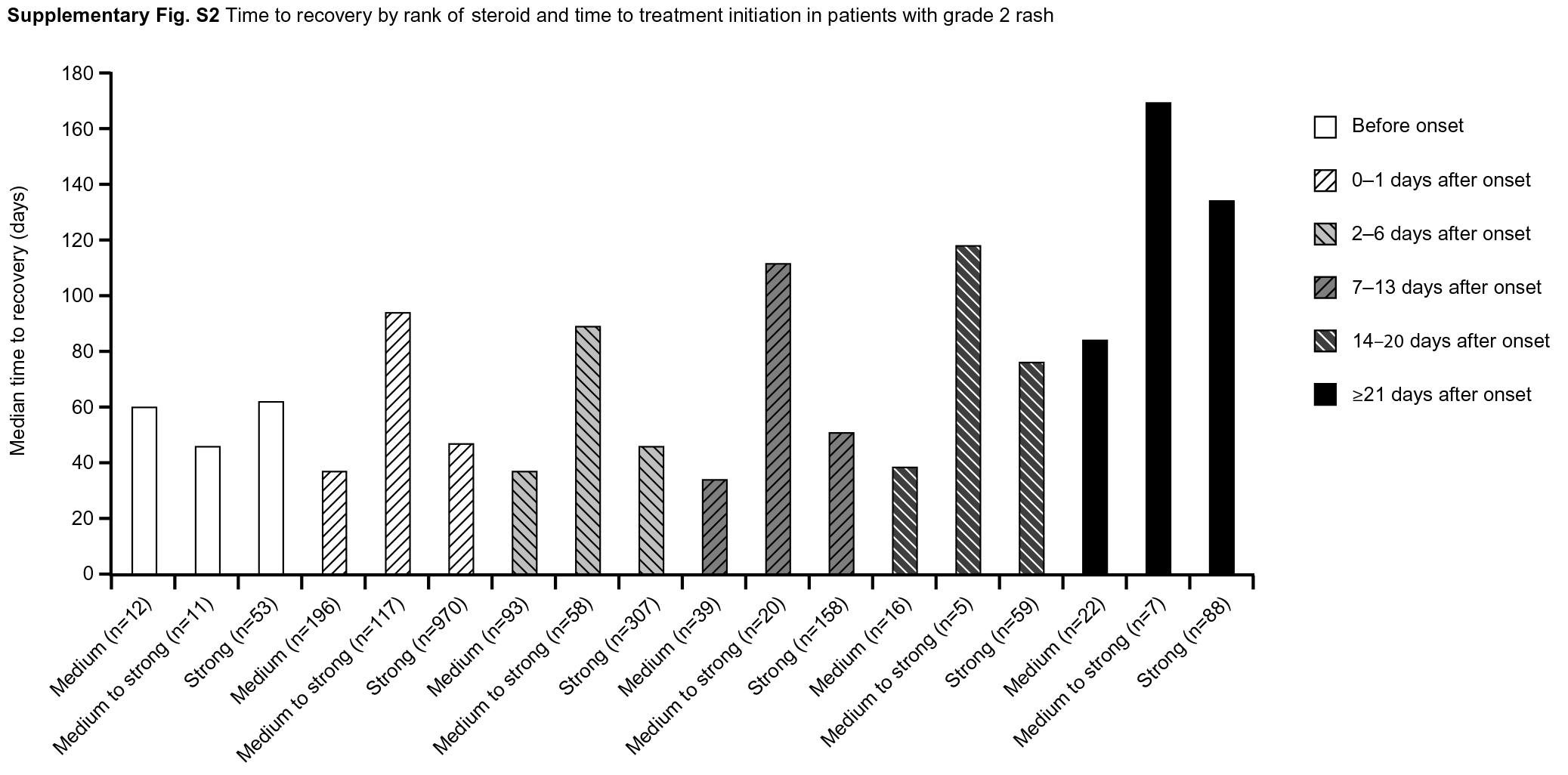

Supplement: Supplementary file 2 — Supplementary Fig. S2 Time to recovery by rank of steroid and time to treatment initiation in patients with grade 2 rash. (TIFF 1237 kb) [file 10147_2015_893_MOESM2_ESM.tif]

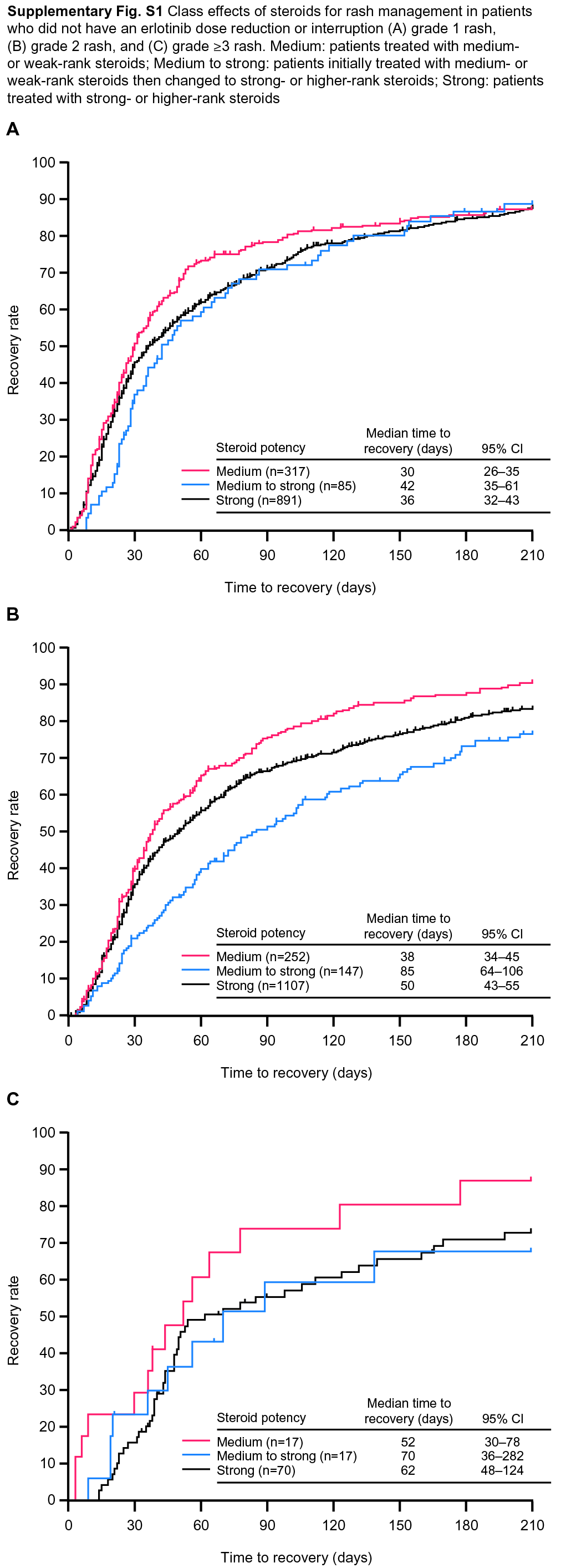

Supplement: Supplementary file 3 — Supplementary material 3 (TIFF 1409 kb) [file 10147_2015_893_MOESM3_ESM.tif]
